# RelB sustains endocrine resistant malignancy: an insight of noncanonical NF-κB pathway into breast Cancer progression

**DOI:** 10.1186/s12964-020-00613-x

**Published:** 2020-08-17

**Authors:** Mei Wang, Yanyan Zhang, Zhi Xu, Peipei Qian, Wenbo Sun, Xiumei Wang, Zhang Jian, Tiansong Xia, Yong Xu, Jinhai Tang

**Affiliations:** 1 Department of General Surgery, The First Affiliated Hospital with Nanjing Medical University, 300 Guangzhou Road, Nanjing, 210029 P. R. China; 2 Jiangsu Cancer Hospital & Jiangsu Institute of Cancer Research & The Affiliated Cancer Hospital of Nanjing Medical University, 42 Baiziting, Nanjing, 210009 P. R. China; 3 Breast Disease Center, The First Affiliated Hospital with Nanjing Medical University, 300 Guangzhou Road, Nanjing, 210029 P. R. China; 4 Jiangsu Key Lab of Cancer Biomarkers, Prevention and Treatment, Nanjing Medical University, Nanjing, 211166 P. R. China; 5 Department of Toxicology and Cancer Biology, University of Kentucky Markey Cancer Center, 1059 VA Dr, Lexington, KY 40513 USA

**Keywords:** RelB; EMT, MMP1, Cancer progression and breast cancer

## Abstract

**Background:**

The activation of the NF-κB pathway plays a crucial role in the progression of breast cancer (BCa) and also involved in endocrine therapy resistance. On the contrary to the canonical NF-κB pathway, the effect of the noncanonical NF-κB pathway in BCa progression remains elusive.

**Methods:**

BCa tumor tissues and the corresponding cell lines were examined to determine the correlation between RelB and the aggressiveness of BCa. RelB was manipulated in BCa cells to examine whether RelB promotes cell proliferation and motility by quantitation of apoptosis, cell cycle, migration, and invasion. RNA-Seq was performed to identify the critical RelB-regulated genes involved in BCa metastasis. Particularly, RelB-regulated MMP1 transcription was verified using luciferase reporter and ChIP assay. Subsequently, the effect of RelB on BCa progression was further validated using BCa mice xenograft models.

**Results:**

RelB uniquely expresses at a high level in aggressive BCa tissues, particularly in triple-negative breast cancer (TNBC). RelB promotes BCa cell proliferation through increasing G1/S transition and/or decreasing apoptosis by upregulation of Cyclin D1 and Bcl-2. Additionally, RelB enhances cell mobility by activating EMT. Importantly, RelB upregulates bone metastatic protein MMP1 expression through binding to an NF-κB enhancer element located at the 5′-flanking region. Accordingly, in vivo functional validation confirmed that RelB deficiency impairs tumor growth in nude mice and inhibits lung metastasis in SCID mice.

**Video abstract**

## Background

According to the latest statistic data, the numbers of new cases and deaths of breast cancer (BCa) accounted for 11.6 and 6.6% of total cancers worldwide [1]. Since the past decade, BCa incidence has been consistently increasing in developing countries such as South America, Asia and Africa [2]. Owing to improved early diagnosis and advanced therapy strategies, the current death rates of BCa have appreciatively decreased in the Western developed countries, including the United States. However, distant-organ metastasis associated with endocrine therapy resistance still remains as a large obstacle to successful control of advanced BCa [3, 4]. To data, BCa patients with distant metastasis at the time of diagnosis appeared to be worse prognosis with a 5-year survival rate of 23.4% [5]. Since metastasis is the main cause of death of BCa patients, the key for improving the BCa survival rate is to accurately evaluate the metastatic potential for the guidance of clinical implementation of the individualized treatment plan. Current therapeutic strategies for BCa mainly consisted of surgical reduction and radiotherapy-based locally therapy with anticancer drugs provided systemic therapy including chemotherapy, endocrine therapy and targeted therapy. However, the comprehensive treatment strategy is still limited and a large number of patients eventually developed to more aggressive malignant forms that are resistant to the most of treatments [6, 7]. Thus, the discovery of new biomarkers for surveillance of the disease progression and novel molecular targets for improving therapeutic efficiency are urgently needed.

Nuclear factor-κb (NF-κB), a family of transcription factors, regulates immune responses and inflammation [8]. It has been well-documented that NF-κB plays a crucial role in BCa endocrine therapy resistance [9]. In particular, NF-κB activation is thought to promote BCa progression toward ER-independent phenotypes, especially for the reoccurrence of triple-negative BCa (TNBC) after hormone treatment [10, 11]. Mammals express five NF-κB members, including NF-κB1 (p50/p105), NF-κB2 (p52/p100), RelA (p65), RelB and c-Rel [12, 13]. Based on the components of the signaling cascade, the NF-κB signaling can be activated via either canonical pathway (p50:RelA) or noncanonical pathway (p52:RelB) [14, 15]. The canonical NF-κB signaling pathway is regarded as the central regulator of the inflammatory response, but it has been extensively studied in the context of hormone disorders, autoimmunity, obesity and cancer [16, 17]. Nevertheless, the molecular basis for the activation of the noncanonical NF-κB pathway a well as its role in cancer progression have been recently receiving increased attention [18].

RelB has been originally identified in B-cells and the RelB-based noncanonical NF-κB signaling has been recognized to impair antigen presentation by DCs, which is associated with skin inflammation and excessive immune cell infiltration to various organs in the past decade [19–21]. Recently, RelB has been implicated in cancer progression, particularly in sex hormone-related cancers including BCa, prostate cancer (PCa) and cervical cancer [22–24]. We have reported that RelB is highly expressed in advanced PCa and enhances radioresistance, speculating that the activation of noncanonical NF-κB pathway contributes to PCa malignancy from androgen receptor (AR)-dependent to AR-independent phenotypes [25, 26]. The present study further demonstrated that RelB highly expresses in advanced BCa, which is necessary for sustaining BCa malignant progression. Mechanistically, RelB promoted BCa cell proliferation, EMT and metastasis through transcriptional upregulation of several oncogenic proteins, including Bcl-2, Cycline D1 and MMP1.

## Materials and methods

### BCa patient tissue samples and immunohistochemistry (IHC)

The First Affiliated Hospital with Nanjing Medical University (Nanjing, China) has collected fresh tumor tissues from BCa patients prior to treatment and noncancerous breast tissues. The Ethics Committee of Nanjing Medical University has approved the study protocol with written informed consents obtained from all the patients participated in this study. A total of 40 BCa cases (including 21 cases with lymphatic metastasis) were selected to examine the correlation between the level of RelB and BCa pathological grade or stage. The tissues were fixed paraffin-embedded slides. The tissue slides were dewaxed by xylene and then ethanol washing off excess liquid. The slides were further rehydrated by rinsing with dH_2_O. For IHC, the slides were soaked in 5% BSA buffer for 1 h and then incubated with 400x diluted primary antibodies within 5% BSA buffer at 4 °C overnight. The slides were washed and then incubated with biotinylated secondary antibody at room temperature for 30 min. After washing the slides with 1x BPS, a DAB Substrate Kit (Cell Signaling Tech., USA) was used to observe immunostaining images under a microscope. The intensity of IHC staining was scored as negative (score 0), weak (1), medium (2), and strong (3). Total cell positivity was scored as the percentage of positive cells, including no positive cell (0), < 25% (1), 25–50% (2), 50–75% (3), and > 75% (4), respectively. The “H-score” was calculated using “∑pi (i + 1)” for all slides, in which “pi” represented the percentage of positive and “i” represented the staining intensity.

### Cell culture and gene manipulation

All the selected human breast cell lines were purchased from the American Type Culture Collection (ATCC, USA) and grown in the recommended media, including normal breast epithelial cell line (MCF-10A), ER-positive BCa cell lines (MCF-7 and T47D), and TNBC cell lines (MDA-MB-231 and BT549). RelB was ectopically expressed in ER-positive cells with low constitutive RelB. Conversely, RelB was knocked out from the TNBC cells using CRISPR-Cas9 approach. Briefly, several gRNAs were designed using the Optimized CRISPR Design Tool [27]. Double-strand oligo DNA for each gRNA was cloned into the EcoRIsite in pGL3-U6 SPsgRNA vector. The construct was co-transfected with 331-sp cas9 plasmid into MDA-MB-231 and BT549 cells. Single-cell colonies were selected using puromycin and neomycin, and further validated by western blots. DNA was extracted from the candidate clones and confirmed by DNA sequencing prior to T7EN1 digestion.

### Cell proliferation

Cell proliferation was determined using cell counting and colony formation assay, respectively. BCa cells were seeded into 96-well plates at 10^3^ cells/well and treated with CCK-8 reagent (Dojindo Mol. Tech., Japan), and cell viability was measured as the optical density at 450 nm. For colony formation assay, BCa cells were seeded into 6-well plates at 200 cells/well and cultured for 2–3 weeks. The cells were fixed in 4% paraformaldehyde for 30 min, stained with 0.5% crystal violet (Beyotime Biotech., China) for 1 h. After rinsing three times with 1xPBS, the cell colonies were photographed and counted.

### Flow cytometry

Flow cytometry was used to analyze apoptosis and cell cycle. For apoptotic cell qualification, 2 × 10^5^ cells were seeded in 6-well plates and cultured for 48 h. The cells were collected and washed three times with cold PBS and then stained with 5 μM annexin V-FITC and 2.5 μM PI (Dojindo Mol. Tech.) in a supplied binding buffer for 15 min at room temperature and dark condition. Apoptotic cells were counted using a BD FACSCalibur flow cytometer (BD Biosciences, USA) and the percentage of apoptotic cells was calculated by the number of preapoptotic and apoptotic cells divided by the amount of total cells. For cell cycle analysis, the cultured cells were detached, washed and fixed with 70% ethanol overnight at 4 °C. The cells were then washed 2 times with cold PBS and treated with 400 μl propidium iodide (BD Biosciences, USA) for 15 min. The cell cycling phases were determined by Flow cytometry.

### Cell motility

Cell movability was analyzed using wound healing assay and transwell assay, respectively. BCa cells were plated into 6-well plates at 3 × 10^5^ cells/well with serum-free DMEM for 12 h. After rinsing with 1 × PBS three times, the cultured cells were separated using pipette tips to create three parallel wounds. The wells were then washed several times with PBS to remove floating cells. The cells were continuatively cultured in the completed media. The wound closure was monitored at 24 and 48 h using a microscope and the wound surface area was quantified. In addition, transwell assay (Corning, USA) was used to quantify cell migration and invasion abilities. For migration assay, the cells were plated into the upper chambers without coated membrane at 5 × 10^4^ cells/well. For invasion assay, the chamber inserts were coated with 50 μl serum-free medium mixed with matrigels (BD Biosci., USA) and solidified at 37 °C for 3 h. The cells were placed into the upper chamber at 10^5^ cells/well. In both assays, the cells were plated in 200 μl serum-free medium in the upper chambers, and 500 μl medium supplemented with 20% FBS was placed into the lower chambers. After culturing the cells for 24 h, the invaded cells on the lower surface were fixed in 4% paraformaldehyde, stained with crystal violet and counted using a microscope.

### RNA sequencing

Total RNA was isolated from BCa cells using a total RNA isolation kit (Invitrogen). mRNA was converted to cDNA libraries and added adapters for sequencing. The procedure of NGS sequencing on Illumina HiSeq platform was conducted by Vazyme Biotech Co., Ltd. (Nanjing, China).

### Chromatin Immunoprecipitation (ChIP)

A ChIP-IT system (Active Motif, USA) was used to quantify RelB binding to the NF-κB enhancer element located at the 5′-flanking region of the human *MMP1* gene. Briefly, chromatin isolated from BCa cells were pulled down using a RelB antibody (Cell Signaling, USA). Unprecipitated chromatin was used as input control and chromatin pulled down by an IgG antibody served as a negative antibody control. The pulled down the enhancer fragment was quantified using a quantitative PCR with the gene-specific primers.

### Western blotting

Cytosolic and nuclear proteins were extracted from cells and tumor tissues using a RIPA lysis buffer containing PMSF and then quantified using a BCA assay kit (KeyGen Biotech., China). The extracted proteins (50–100 μg) were separated on SDS-PAGE gels and then transferred to PVDF membranes. The membranes were subsequently incubated overnight at 4 °C with the primary antibodies against RelB, Bcl-2, Cyclin D1 and β-actin (Santa Cruz Biotech., USA); against ER, E-cadherin, Vimentin, Snail 1, Slug, Twist 1 (Cell Signaling Tech., USA). Thereafter, the membranes were washed three times with TBST buffer and incubated at room temperature for 2 h with HRP-conjugated secondary antibody (Santa Cruze Biotech.). The immunoblots were visualized using an enhanced chemiluminescence detection system (Bio-Rad, USA). The intensities of blots were quantified using Quantity One software and protein expression was normalized by loading controls such as β-actin and GAPDH.

### Animal experiment

The effects of RelB on tumorigenesis and metastasis were validated using BCa cells bearing mouse xenograft tumor experimental models. All animal studies were conducted according to the Institutional Animal Care and Use approved by the Research Committee of Nanjing Medical University (No. IACUC-1711030). Five-week-old female BALB/c athymic nude mice (Beijing Vital River Lab Animal Tech. Co., Ltd., China) were used for studying tumor growth and five-week-old female SCID mice (Nanjing Medical University, China) were used for studying tumor metastasis, respectively. For the tumor growth experiment, 5 × 10^6^ BCa cells were subcutaneously implanted into the right axilla of mice. Tumor volume was measured using digital calipers every other day and calculated using a standard formula (V = 0.52 × AB^2^, A and B represent the diagonal tumor lengths). The mice were executed when tumor volume reached to 2000 mm^3^ and tumor tissues were removed. For tumor metastasis study, 10^6^ BCa cells were injected into mice through tail vein and assessed for lung metastasis. The mice were sacrificed at 6 weeks and the number of metastatic lung nodules was counted.

### Usage of TCGA database

The TCGA BCa dataset was analyzed to assess the association of RelA or RelB expression with BCa occurrence and the correlation between the mRNA level of RelA or RelB and ER-negative BCa patient survival rate.

### Statistical analysis

Data were presented as the mean ± standard deviation (SD) from at least three replicates. Significant differences between the experimental groups were analyzed by unpaired Student’s t-test. One-way analysis of variance (ANOVA) followed by Dunnett’s or Bonferroni’s multiple comparison test was performed using Prism (GraphPad, San Diego, USA). Statistical significance was accepted at *P* < 0.05.

Additional materials and methods are in Additional file 1**.**

## Results

### RelB is correlated with BCa aggressiveness

**Fig. 1:**
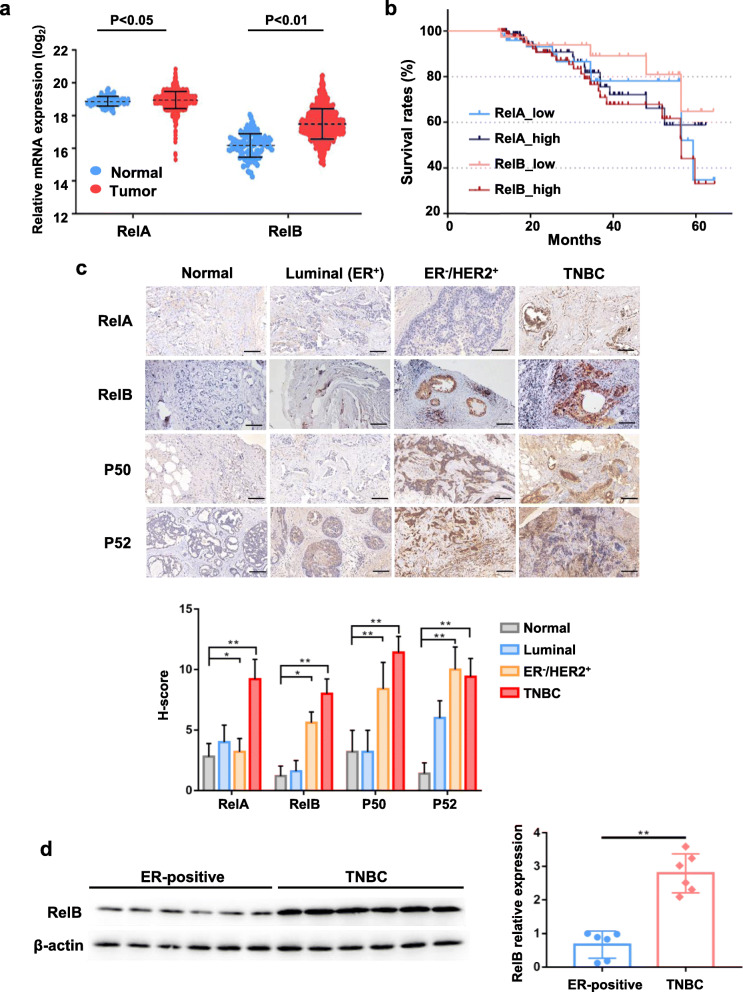
The correlation between RelB and aggressiveness of BCa. **a**, **b** TCGA database (GEO accession: GSE11121) was analyzed to examine the correlation between mRNA expression profiles of RelA and RelB and BCa occurrence and over survival of patients. **c** BCa tumor tissues with different pathological grades and normal breast tissues were screened by IHC using NF-κB antibodies. The Luminal group was characterized as ER-positive and HER2-negative low-grade genotype; HER2^+^ group was assessed as a middle-grade genotype with high tumor growth; TNBC group was determined as a high grade. **d** The levels of RelB in TNBC tumors vs. ER-positive BCa tumors were quantified, β-actin-normalized images were plotted

We analyzed the BCa cohort in TCGA database to examine whether the expression level of RelB is correlated to BCa progression and patient survival. The statistical analytic data indicated that the mRNA levels of RelA and RelB in tumor tissues were higher than their levels in normal breast tissues, particularly the difference in RelB levels appeared to be highly associated with BCa progression (Fig. 1a). Consistently, the expression of RelA and RelB was also associated with the overall survival of ER-negative BCa patients. Notable, compared to RelA, the high level of RelB led to lower survival rates (Fig. 1b). To assess the correlation between NF-κB and BCa, normal breast tissues and tumor tissues from the selected BCa patients with different stages were analyzed by IHC (Additional file 2 Table S1). Compared to the normal breast tissue control, the levels of four members of the NF-κB family increase in BCa tumor tissues, especially in the ER-negative phenotype. Notably, the expression of RelB is apparently correlated to the aggressiveness of BCa (Fig. 1c). Subsequently, we further examined the levels of RelB in TNBC tissues vs. ER-positive BCa tissues by western blots. As expected, the high levels of RelB were detected in all the TNBC tissues, suggesting the RelB is inversely related to ER in BCa (Fig. 1d).

**Fig. 2:**
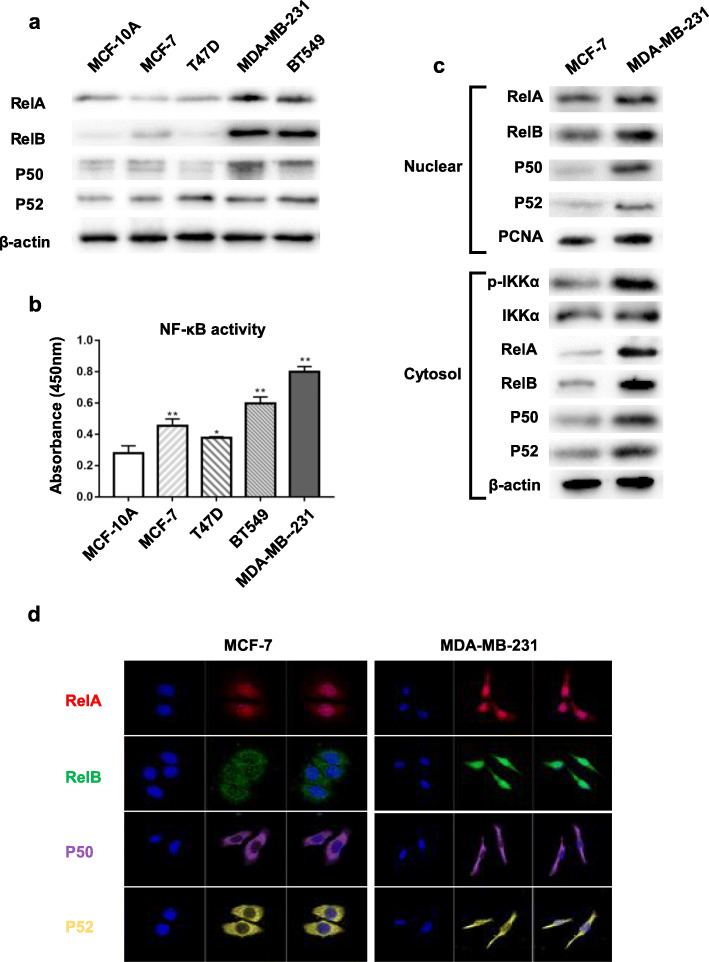
The NF-κB activation in BCa cell lines. **a** Several corresponding cell lines were selected to confirm the relationship between NF-κB proteins and the aggressiveness of BCa cells. **b** The relative NF-κB activity was quantified in the BCa cell lines. **c** Cytoplasmic and nuclear levels of NF-κB proteins in ER-positive MCF-7 and TNBC MDA-MB-231 cells were quantified. Accordingly, the NF-κB upstream phosphorylated IKKα levels in the two cell lines were measured. **d** The cellular distribution of the NF-κB proteins in the two cell lines were further examined using a confocal microscope

Furthermore, several BCa cell lines correspondent to the different stages of BCa progression vs. a normal breast epithelial cell line MCF-10A were examined. Consistently, the high constitutive levels of the NF-κB members were detected in BCa cell lines, particularly RelB uniquely expressed at high levels in TNBC cell lines (Fig. 2a). Accordingly, the NF-κB activity was consistently elevated in BCa cells, especially in TNBC cells (Fig. 2b). Furthermore, ER-positive MCF-7 cells vs. triple-negative MDA-MB-231 cells were used to measure cytoplasmic and nuclear levels of the NF-κB members. As expected, all the members highly increased in MDA-MB-231 cells compared to MCF-7 cells. Importantly, the NF-κB upstream IKKα and its phosphorylated levels also heighten in TNBC cells (Fig. 2c). The distribution of the NF-κB members was further confirmed by confocal microscope (Fig. 2d). Taken together, these results indicated that the high level of RelB is associated with aggressiveness of BCa, suggesting that RelB may contribute to the advanced BCa.

### RelB promotes BCa cell proliferation

**Fig. 3:**
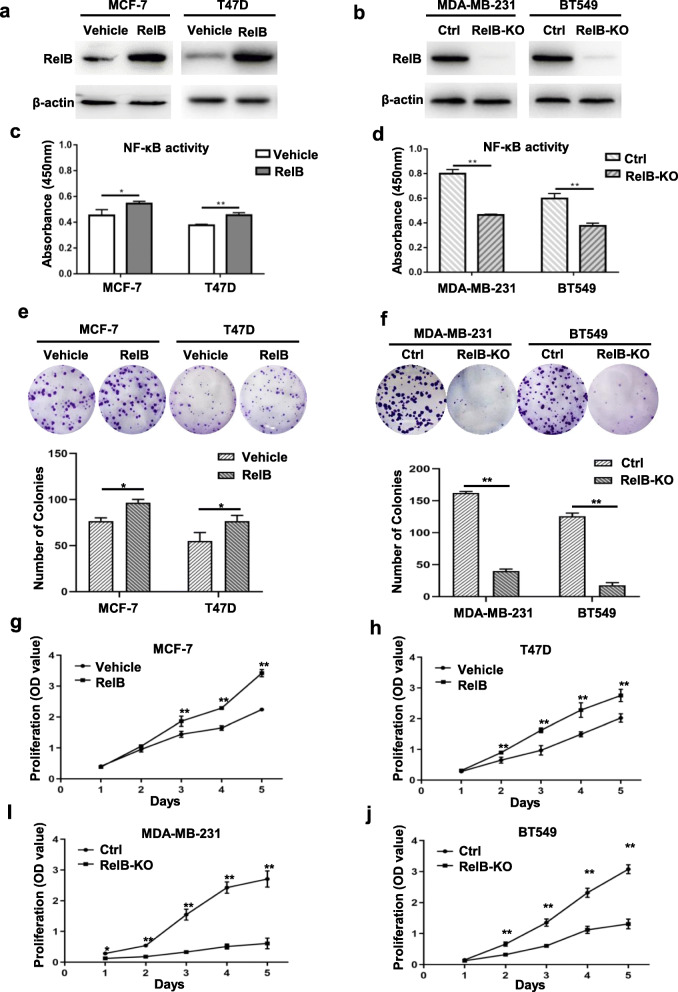
Determination of RelB-enhanced BCa cell survival and growth. **a** RelB was ectopically expressed in ER-positive BCa cells by stably transfected a human RelB cDNA construct. **b** RelB was knocked out in TNBC cells using a CRISPR-Cas9 gene edition system. **c, d** The relative NF-κB activity was quantified in RelB-manipulated BCa cells. **e, f** The survival rates in the RelB-manipulated BCa cells were examined by colony formation assay. **g-j** The proliferation rates in the RelB-manipulated BCa cells were quantified by CCK-8 assay. Mean ± SD was representative of three independent experiments carried out in duplication. *(*P* < 0.05), **(*P* < 0.01) show the significances between two groups as indicated

To investigate the potential mechanism by which RelB regulates downstream gene expression involved in BCa progression, RelB was ectopically expressed into MCF-7 and T47D cells with low levels of constitutive RelB (Fig. 3a). In parallel, RelB was knocked out from TNBC cells (MDA-MB-231 and BT549) using a CRISPR-Cas9 gene edition system (Additional file 3, Figure S1; Fig. 3b). The NF-κB activity was slightly elevated in the RelB-overexpressed ER-negative cells, but significantly reduced in RelB-knocked out TNBC cells (Fig. 3c and d). Consistently, the elevated RelB in ER-positive cells led to increased cell colony number and the cell proliferation rate (Fig. 3e, g and i). Conversely, cell colony formation and the cell proliferation rate significantly decreased in RelB-knocked out TNBC cells (Fig. 3f, h and j).

### RelB promotes G1/S transition and inhibits apoptosis

**Fig. 4:**
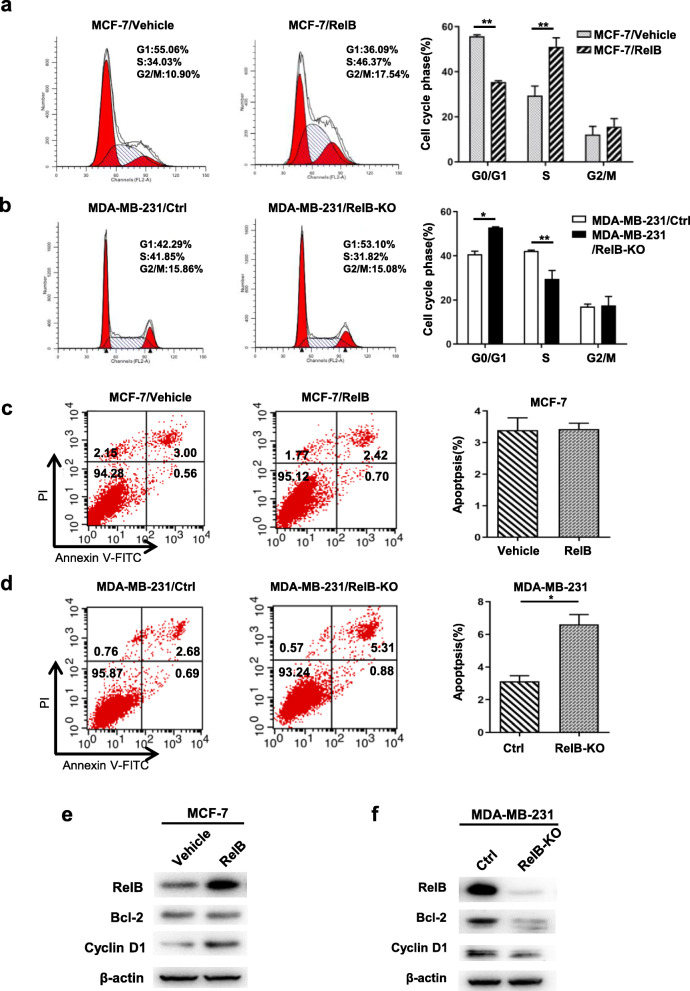
The effect of RelB on cell cycle and apoptosis. **a, b** The cell cycling process in RelB-manipulated BCa cells was analyzed using flow cytometry. **c, d** Preapoptotic and apoptotic cells in the RelB-manipulated BCa cells were quantified by flow cytometry. **e, f** The levels of Bcl-2 and Cyclin D1 proteins were measured by western blots. Statistical significance between two groups as described in Fig. 3

To elucidate the role of RelB in BCa cell proliferation, the cell cycling process and the cell apoptotic rate were analyzed by flow cytometry. The enforced expression of RelB in MCF-7 cells resulted in decreasing G1 phase but increasing S and G2/M phases, which enhances the acceleration of cell division (Fig. 4a). In contrast, knock out of RelB in MDA-MB-231 cells led to an increasing G1 phase but decreasing S phase (Fig. 4b). Furthermore, apoptosis was examined in the RelB-manipulated BCa cells. Although the elevated RelB in MCF-7 cells unlikely changed the cell apoptotic rate, the depletion of RelB in MDA-MB-231 cells obviously increased apoptotic cell rates (Fig. 4c and d). Consistently, the overexpression of RelB in MCF-7 cells resulted in upregulation of Cyclin D1, but no effect on Bcl-2 expression (Fig. 4e). Additionally, knock out of RelB in MDA-MB-231 cells led to suppression of Cyclin D1 and Bcl-2, suggesting that RelB sustains TNBC cell growth via activation of prosurvival and antiapoptotic pathways (Fig. 4f). Moreover, RelB depletion led to increases in p21 and p27, but decreases in c-Myc, Cyclin E1 and Bcl-xL (Additional file 4, Table S2; Additional file 5, Figure S2).

### RelB enhances BCa cell mobility by enhancing EMT

It has been widely recognized that BCa progression is generally through the ER-dependent phenotype to ER-independent malignancy. Importantly, emerging evidence demonstrated the inverse relationship between ER and NF-κB in BCa cells [28]. Consistently, the expression of RelB appeared to be inversely correlated to ER levels in the tested cell lines (Additional file 6, Figure S3a). Furthermore, ER elements-driven luciferase reporter gene expression construct was constructed to examine whether RelB is able to regulate the ER response. Expectedly, the ER-reporter response was remarkably reduced due to the increased RelB in MCF-7 cells, suggesting that RelB negatively regulates ER signaling (Additional file 6, Figure S3b).

**Fig. 5:**
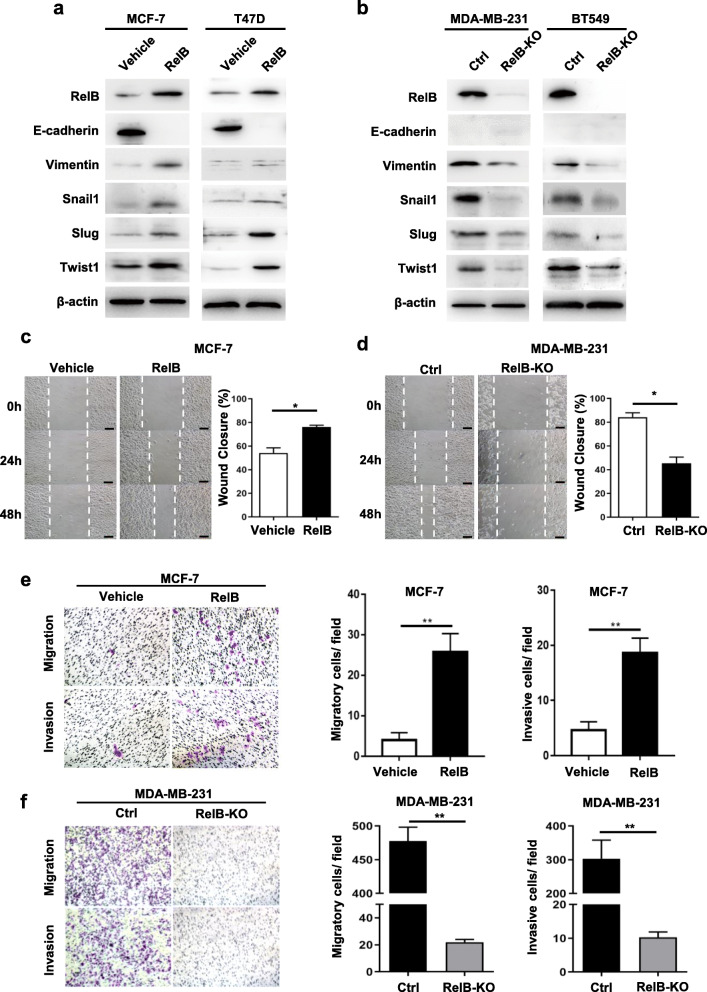
The effect of RelB on EMT. **a, b** The EMT-associated markers in RelB-manipulated BCa cells were measured by western blots. **c, d** The cell wound healing abilities were analyzed. **e, f** The cell a migration and invasion capacities were examined using a transwell assay. Statistical significance between two groups as described in Fig. 3

Furthermore, to testify whether RelB contributes to estrogen-deprived BCa progression, the effect of RelB on the metastatic ability of BCa cells was examined. Since the EMT process has been well documented in BCa metastasis, several EMT markers were measured in our panel of BCa cell lines. The elevated RelB in ER-positive cells led to decreasing E-cadherin but increasing Snail I, Slug I, Twist I and Vimentin (Fig. 5a). In parallel, knock out of RelB in TNBC cells resulted in decreasing those EMT markers, while E-cadherin was not detectable in TNBC cells (Fig. 5b). Accordingly, the effect of RelB on the cell motility was analyzed by quantifying cell capacities of wound healing, migration and invasion. As expected, the enforced expression of RelB in MCF-7 cells significantly enhanced the cell capacity for wound recovery compared to a vehicle control (Fig. 5c). Consistently, the elevated RelB in MCF-7 cells also resulted in enhancing cell migration and invasion (Fig. 5e). Subsequently, knock out of RelB in MDA-MB-231 cells led to decreases in those capacities (Fig. 5d and f). These results suggest that RelB promotes ER-independent BCa progression mainly through activating EMT process.

### RelB upregulates bone metastasis associated protein, MMP1

**Fig. 6:**
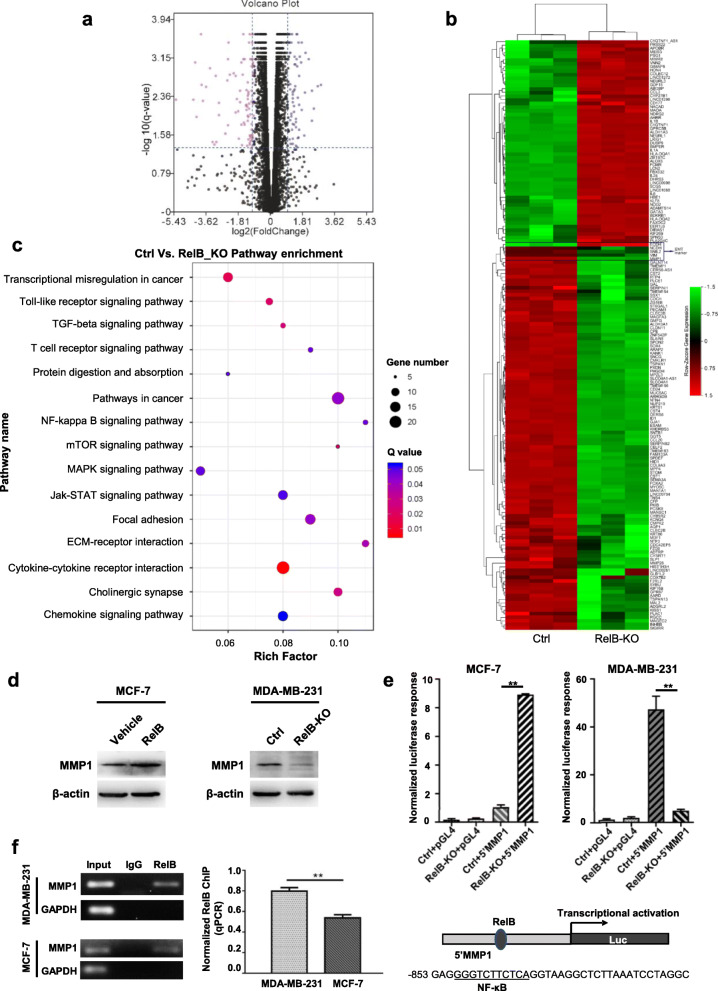
RelB-mediated transcriptional regulation. mRNA expression profiles in the RelB-knocked out MDA-MB-231 cell vs. the parent cell were examined using a RNA-Seq platform. **a** The distribution of mRNA profiles in the RelB-deprived cell was illustrated. **b** Cluster analysis of RNA-Seq data indicates up/down-regulated mRNA profiles (heatmap). **c** KEGG pathway enrichment analysis was performed to assess EMT/metastasis relating mRNA profiles. **d** The protein level of MMP1 was quantified in the RelB-manipulated BCa cells. **e** A 5′-flanking region of the human *MMP1* gene (5’MMP1) containing the NF-κB element (underline) and the core promoter region was cloned in pGL4 vector to drive the luciferase reporter gene expression as illustrated. The enhancer activity modulated by RelB in BCa cells was estimated by normalized reporter responses. **f** A small DNA fragment containing the NF-κB element was pulled down from chromatins using a RelB antibody and then quantified by PCR. IgG serves as a negative antibody control. Statistical significance between two groups as described in Fig. 3

To identify the important RelB-regulated genes involved in BCa metastasis, we applied RNA-Seq analysis using total RNA isolated from RelB-knocked out MDA-MB-231 cells vs. the parent cells. The results indicated that the mRNA expression profiles of numerous genes were altered in the RelB-knocked out cells (Fig. 6a and b). Importantly, multiple genes relating to metastatic signaling pathways were downregulated (Fig. 6c and Table 1). Among of them, MMP1, an activator in BCa bone metastasis [29], was highly related to the level of RelB. To assess the effect of RelB on MMP1 expression, we verified the expression level of MMP1 increased in RelB-overexpressed MCF-7 cells, but decreased in RelB-knocked out MDA-MB-231 cells, suggesting that RelB may directly regulate the *MMP1* gene (Fig. 6d).

**Table 1:** mRNA expression profiles in MDA-MB-231 cells

Gene name	Ctrl	RelB-KO	log_2_(RelB-KO/Ctrl)	up-or-down	*P* value
VIM	1105.77	968.23	−0.1916	down	0.0411
CDH11	14.2157	5.1362	−1.4687	down	5.00E-05
ZEB1	8.9261	4.7115	−0.9219	down	5.00E-05
SNAI1	0.6309	0.474	−0.4124	down	0.1663
MMP1	108.85	7.6109	−3.8382	down	5.00E-05
MMP9	0.7118	0.3904	−0.8663	down	0.0037
MMP28	1.134	0.2263	−2.3250	down	0.0008
JUN	53.297	154.57	1.5361	up	0.0001
Hsp70	1.5236	3.1771	1.0602	up	0.0001
DMBT1	0.9316	1.9726	1.0822	up	5.00E-05
SMAD2	7.1095	7.0939	−0.0032	unchanged	0.9734

*VIM* vimentin, *CDH11* cadherin 11, *ZEB1* zinc finger E-box-binding homeobox 1, *SNAI1* snail1, *MMP1* matrix metallopeptidase 1, *MMP9* matrix metallopeptidase 9, *MMP28* matrix metallopeptidase 28, *JUN* Jun proto-oncogene (AP-1 transcription factor), *Hsp70* heat shock protein 70, *DMBT1* deleted in malignant brain tumor 1 (tumor suppressor), *SMAD2* a member in TGF-B/Smad signaling family

To elucidate how RelB regulates MMP1 expression, we identified a putative NF-κB element located in the 5′-finking region of the human *MMP1* gene. For verifying this NF-κB element functional response to transcriptional activation by RelB, a 5′-flanking region containing the NF-κB enhancer element and the core promoter in the human *MMP1* gene was cloned into a pGL4 vector to drive the *luciferase* reporter gene expression. Compared to the basic vector control, the reporter gene response driven by the *MMP1* enhancer highly increased in RelB-overexpressed MCF-7 cells, but significantly decreased in RelB-knocked out MDA-MB-231 cells (Fig. 6e). Moreover, to confirm that RelB-mediated transcriptional activation through binding to the NF-κB enhancer element located at the 5′-flanking region of the *MMP1* gene, a 240-bp fragment containing the NF-κB enhancer element was pulled down using a specific RelB antibody and amplified using PCR. The amount of pulled-down DNA fragment from MCF-7 cell chromatin was less than the level from MDA-MB-231 cell chromatin (Fig. 6f).

Furthermore, to validate whether the increased MMP1 can further promote EMT, MMP1 was manipulated by overexpressing in MCF-7 cells but silencing in MDA-MB-231 cells. The elevated MMP1 in MCF-7 cells led to a slight decrease in E-cadherin but increases in Snail 1, Twist1 and Vimentin. In contrast, the silence of MMP1 in MDA-MB-231 cells resulted in decreasing Snail 1, Twist 1, Slug and Vimentin, regardless of the undetectable of E-cadherin in the cells (Additional file 7, Figure S4a). In addition, to determine the functional contribution of MMP1 to EMT under the RelB regulation, MMP1 was either ectopically expressed in RelB-knocked out MBA-MB-231 cells or was silenced in RelB-overexpressed MCF-7 cells. Intriguingly, the elevated MMP1 in RelB-knocked out MBA-MB-231cells resulted in partially restored the EMT proteins, while the silence of MMP1 in RelB-overexpressed MCF-7 cells was able to abrogate the elevated EMT proteins except for no effect on Twist 1 expression (Additional file 7, Figure S4b).

### RelB-mediated BCa progression was validated in mice

**Fig. 7:**
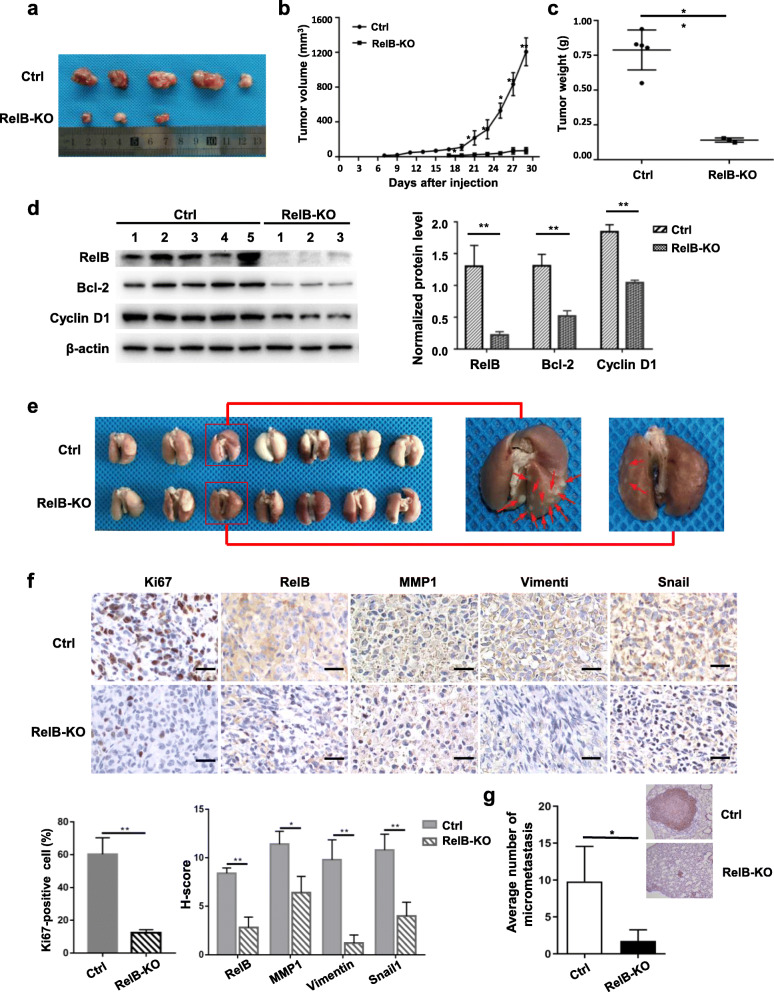
Validation of RelB-enhanced tumor growth and metastasis in mice. **a** RelB-knocked out MDA-MB-231 and the parent cells were subcutaneously injected into the right axilla of female nude mice and allowed to form tumors. Thirty days after BCa cell implantation, tumor tissues were removed out prior to the execution. The excised tumor tissues were photographed. Two out of five mice injected with RelB-knocked out cells didn’t form the tumor. **b, c** Tumor growth rates and the excised tumor weights were measured and plotted. **d** Total proteins were extracted from the tumor tissues and immunoblotted with the relating antibodies, β-actin served as an internal control. **e** Female SCID mice were injected with RelB-knocked out MDA-MB-231 and the parent cells through the tail vein. After 6 weeks, the mice were sacrificed and lung metastasis was examined. The excised lung-metastasized tumors indicated by arrows were photographed. **f** The metastatic lung tissues were screened by IHC with relative antibodies incorporated with Ki67 staining. The relative images were plotted. **g** H&E staining was applied to conform the lung metastatic tissues and the numbers of micrometastasis in the two groups were plotted. Statistical significance between two groups as described in Fig. 3

Two mouse xenograft tumor experimental models were used to validate the effects of RelB on tumor growth and metastasis in vivo. RelB-knocked out MDA-MB-231 cells and the parent cells were subcutaneously injected into the front leg area of nude mice. One week after injection, tumors were formed and continuously grew to reach the maximum volume (2000 mm^3^). Compared to injection of the control cells, tumor formation by the RelB-knocked out cells was approximately 1 week late. 1 month after injection, the tumor volumes in the control group reached the maximal volume. However, the tumor growing speed and tumor weight were remarkably reduced in the group injected with the RelB-knocked out cells (Fig. 7a-c). According to RelB-knocked out, Bcl-2 and Cycline D1 in the tumor tissues were significantly reduced (Fig. 7d), which just reflected to in vitro results as shown in Fig. 4.

Furthermore, the reduction in the metastatic capacity of the RelB-knocked out MDA-MB-231 cells was validated by injecting the cells through tail vents of SCID mice. Six weeks after the injection, the tumor occurred on the lung of mice injected with the control cells. However, the incidence of metastatic lung tumors was dramatically reduced in the RelB-knocked out group (Fig. 7e). The excised tumor tissues were analyzed by IHC analysis using the relative antibodies. Compared to the control group, tumor aggressiveness stain Ki67 was significantly reduced in the RelB-knocked out group. Consistently, the levels of MMP1 and EMT markers were also reduced in RelB-knocked out group (Fig. 7f). Additionally, tumor images by hematoxylin and eosin (H&E) staining confirmed that the metastasized tumors were obviously reduced in the RelB-knocked out group compared to the control group (Fig. 7g). It should be noted that the detection of low levels of RelB in the RelB-knocked out group reflected the constitutive mouse RelB. Additionally, the enforced expression RelB in MCF-7 cells was still insufficient to tumor metastasize to the lung. Altogether, these results suggest the noncanonical NF-κB pathway may serve as a major contributor to the progression of malignant BCa.

## Discussion

It has been widely recognized that BCa is initialed from ER-dependent phenotype and further promoted to ER-independent malignancy, as per the consequence of cell signaling alteration during this progression is necessary for sustaining malignant development at the ER-negative condition [30]. In this regard, NF-κB, a well-known signal pathway, has been manifested to play a pivotal role in cancer progression, therapeutic resistance and reoccurrence [31–34]. A number of studies have demonstrated that the RelA-based canonical NF-κB pathway is essential for tumorigenesis [35, 36]. Nevertheless, the RelB-based noncanonical NF-κB pathway in cancer progression remains to be elucidated. Wang et al. have shown that RelB is inversely associated with ER in BCa cells and that mechanistically RelB upregulates Blimp1, a transcriptional repressor of ERα [12]. Importantly, it has been noted that RelA can upregulate RelB expression, which is necessary for sustaining the high NF-κB activity for metastasis and therapeutic resistance [37, 38].. In addition, compared to RelA:p50 dimer in the activation of canonical NF-κB pathway, both RelB:p52 and RelB:p50 dimers can activate the noncanonical NF-κB pathway, suggesting that RelB is more feasible for sustaining the NF-κB activity [39]. We have previously reported that RelB is highly expressed in AR-negative advanced PCa cells and silence of RelB sensitized the cells to radiation [25, 26]. The present study demonstrates that the high level of constitutive RelB is necessary for BCa progression by enhancing both tumor growth and metastases. Altogether, the insights from these studies suggest that the activation noncanonical NF-κB pathway may be a common issue in BCa and PCa, which mainly supports malignant progression in response to hormone deprived microenvironment.

Numerous studies demonstrated that the activation of the ER signal pathway is essential for tumorigenesis of BCa, and therefore inhibition of ER resulted in suppression of tumor growth, especially for the early stages of BCa [40, 41]. In the clinic, ER-positive BCa patients treated with antiestrogen drugs like tamoxifen generally have received better therapeutic results. However, the drug-resistant malignant types inevitably relapsed after 1–2 year treatment [23]. Similarly, AR-targeted treatment failed to treat PCa when more aggressive AR-independent forms have been developed [42]. Intriguingly, the activation of NF-κB signal pathway has sequentially emerged to sustain BCa/PCa progression followed by the decline of ER/AR signal signaling. In this regard, the present study showed that the high level of RelB promotes BCa cell proliferation and metastatic capacity. Consequently, the depletion of RelB in BCa cells resulted in decreasing BCa progression in vitro and in vivo.

Although the 5-year survival rate of BCa has been consistently increasing in the past decade, distant-organ metastasis associated with multidrug resistance is still the main cause of BCa death [43–45]. Virtually, ER-positive BCa patients were routinely treated with ER-targeted drugs and have received better therapeutic outcomes. Unfortunately, many BCa patients ultimately develop ER-independent forms and metastasize different organs, especially BCa bone and brain metastases have become big challenges for BCa comprehensive treatment [46, 47]. EMT, as a major metastatic phenotype, is thought to play a critical role in the development of malignant BCa [48, 49]. The present study has not merely observed RelB enhancing tumor growth, but more importantly discovered RelB enhancing the EMT process. Migration and invasion capabilities of BCa cells can be modulated by manipulation of RelB expression in the cells, indicating that RelB is a positive factor for promoting BCa metastasis. The observation was further supported by the evidence that RelB up-regulates Snail, Slug, Twist and Vimentin, but suppresses E-cadherin.

Increasing downstream functional proteins have been identified to be deregulated in EMT-associated BCa metastasis. Interestingly, a variety of factors relevant to calcium metabolism were apparently altered. MMPs function in the breakdown of extracellular matrix to enhance mesenchymal dissemination [50]. It has been reported that calcium-binding protein S100A4 cooperated with MMP1 enhances BCa bone metastasis [51]. Consistently, the present study demonstrated that RelB transcriptionally up-regulates MMP1 expression in advanced BCa. Knock out of RelB resulted in reducing BCa metastasis partially due to the downregulation of MMP1.

**Fig. 8:**
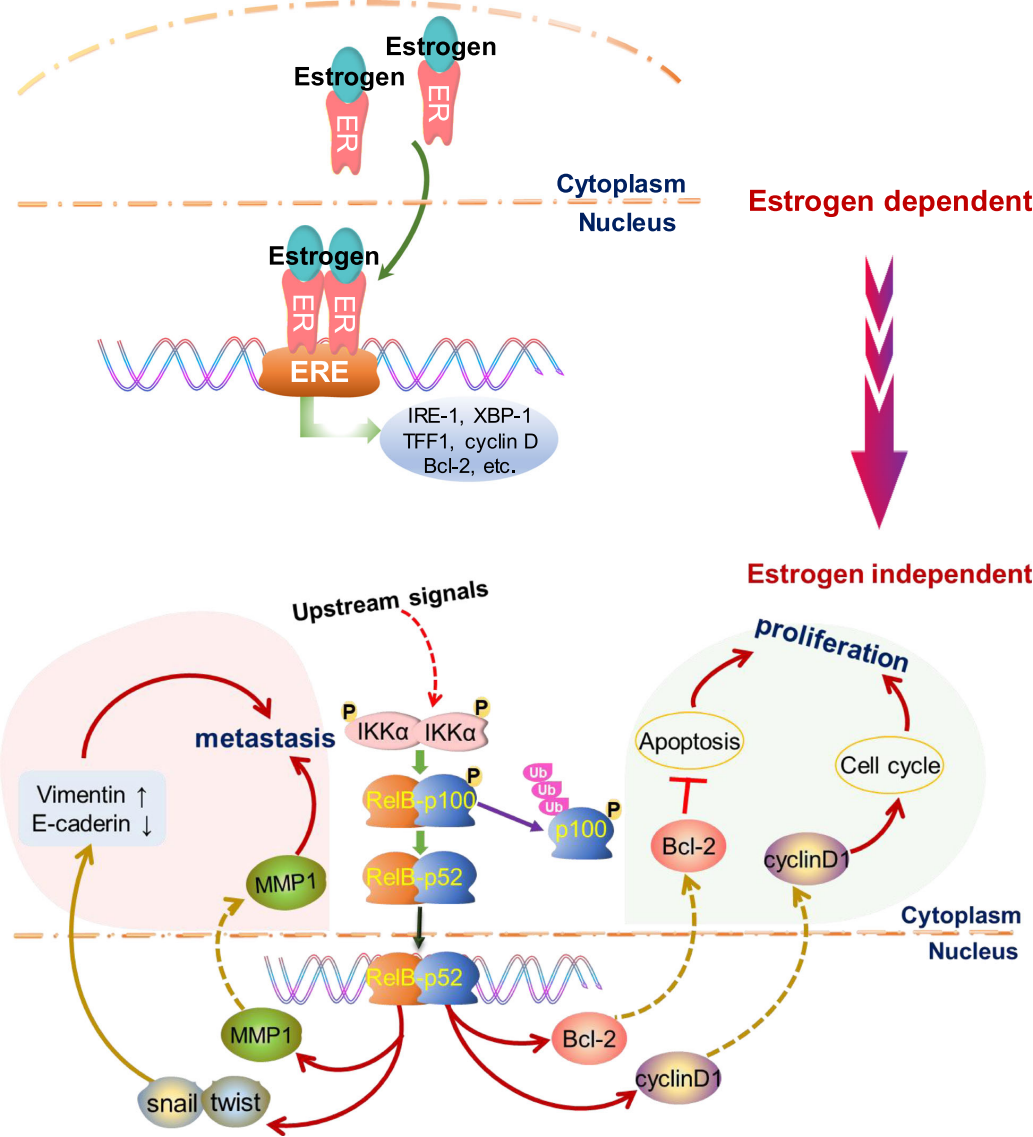
Schematic presentation of the suggested mechanistic integration involved in BCa endocrine-resistant malignancy. In the early stages of BCa, the ER-responsive signal pathway initiates tumorigenesis and promotes local tumor growth. Ultimately, the activation of the noncanonical NF-κB signal pathway predominantly substitutes ER function to sustain BCa metastasis at the estrogen-deprived condition

In summary, the present study revealed that RelB-based NF-κB noncanonical pathway promotes malignant BCa development. Notably, the elevated RelB in ER-positive BCa cells resulted in decreasing ER expression, which further enhanced ER-independent proliferation and promoted the EMT process. Mechanistically, RelB enhances BCa cell survival and proliferation mainly through the upregulation of Bcl-2 and CyclinD1. Moreover, RelB also increases EMT-associated BCa metastasis, at least in part, due to the upregulation of MMP1. The mechanism by which RelB regulates BCa progression as illustrated in Fig. 8. The finding from this study may provide a potential therapeutic approach to control advanced BCa by suppression of the RelB-based noncanonical NF-κB pathway.

## Conclusions

In addition to well-documented RelA-based canonical NF-κB pathway in cancer development, the present study demonstrates that the RelB-based noncanonical NF-κB pathway plays a crucial role in advanced BCa as followed by ER functional decline.

## Supplementary information

**Additional file 1.**

**Additional file 2.**

**Additional file 3.**

**Additional file 4.**

**Additional file 5.**

**Additional file 6.**

**Additional file 7.**

## Data Availability

All materials made and datasets analyzed in this study are available for reasonable requests to the corresponding author, Dr. Yong Xu.

## Abbreviations

BCa: Breast cancer
TNBC: Three negative breast cancer
ER: Estrogen receptor
ERE: ER element
PCa: Prostate cancer
AR: Androgen receptor
TCGA: The Cancer Genome Atlas
NF-κB: Nuclear factor-κb
CDKs: Cyclin-dependent kinases
EMT: Epithelial-to-mesenchymal transition
MMP1: Matrix metalloproteinase-1
ChIP: Chromatin Immunoprecipitation
RT-qPCR: Revers transcription quantitative PCR
