# Supplementary material for: RelB sustains endocrine resistant malignancy: an insight of noncanonical NF-κB pathway into breast Cancer progression
Source: Cell Commun Signal. 2020 Aug 17;18:128. doi: 10.1186/s12964-020-00613-x (PMC7430126; doi:10.1186/s12964-020-00613-x)
Supplement: Supplementary file 2 — Additional file 1. [file 12964_2020_613_MOESM2_ESM.pdf]

Additional Files

Additional file 1. Additional materials and methods:

Gene manipulation

To silence MMP1 in TNBC cells, three siRNA duplexes targeting MMP1 and a scramble control siRNA duplex (RiboBio Co., Ltd., China) were transiently transfected into the cells using Lipofectamine (Invitrogen, USA).

Luciferase reporter assay

To examine whether RelB regulates ER response, three copies of ER elements were cloned into SV40P/pGL3 vector and transiently transfected into RelB-overexpressed BCa cells. In addition, to examine whether RelB directly upregulates transcription of the human *MMP1* gene, a 5'-flanking region of the gene (-2000 To 94) was cloned into pGL4 vector to drive the *Firefly luciferase* reporter gene expression. After cell transfection, the luciferase activity was quantified by the Dual Luciferase Assay System (Promega, USA) using a luminometer (Berthold Tech., Germany). The effect of RelB on the transcriptional regulation was estimated by firefly luciferase activity normalized renilla luciferase activity.

RT-qPCR

Total RNA was isolated from BCa cells using TRIzol reagent (Invitrogen) and converted to cDNA using a PrimeScript RT reagent kit (Takara Biomed. Tech. Co., Ltd., Japan). RT-qPCR for target genes was performed using SYBR Premix Ex Taq DNA polymerase II with a LightCycle System (Roche, USA). The mRNA levels of the target genes were estimated by normalizing with β-actin. Sequences of PCR primers for multiple mRNA are listed in the Additional file 4, Table S2.
