# Supplementary material for: RelB sustains endocrine resistant malignancy: an insight of noncanonical NF-κB pathway into breast Cancer progression
Source: Cell Commun Signal. 2020 Aug 17;18:128. doi: 10.1186/s12964-020-00613-x (PMC7430126; doi:10.1186/s12964-020-00613-x)
Supplement: Supplementary file 3 — Additional file 2. [file 12964_2020_613_MOESM3_ESM.pdf]

Additional file 2. Table S1:

Clinicopathological characteristics of BC patients enrolled in this study

Variable		n=40	
		No. of cases	%
Age (years)			
	<50	14	35
	≥50	26	65
Tumor size(cm)			
	<2	23	57.5
	≥2	17	42.5
Lymph node infiltrated			
	No	19	47.5
	Yes	21	52.5
pTNM stage			
	Stage I/II	18	45
	Stage III/IV	22	55
Pathological grade			
	G1	12	30
	G2	17	42.5
	G3	11	27.5
ER status			
	Negative	21	52.5
	Positive	19	47.5
PR status			
	Negative	22	55
	Positive	18	45
Her-2 status			
	Negative	17	42.5
	Positive	23	57.5
