# Supplementary material for: RelB sustains endocrine resistant malignancy: an insight of noncanonical NF-κB pathway into breast Cancer progression
Source: Cell Commun Signal. 2020 Aug 17;18:128. doi: 10.1186/s12964-020-00613-x (PMC7430126; doi:10.1186/s12964-020-00613-x)
Supplement: Supplementary file 4 — Additional file 3. [file 12964_2020_613_MOESM4_ESM.pdf]

Additional file 3. Figure S1:

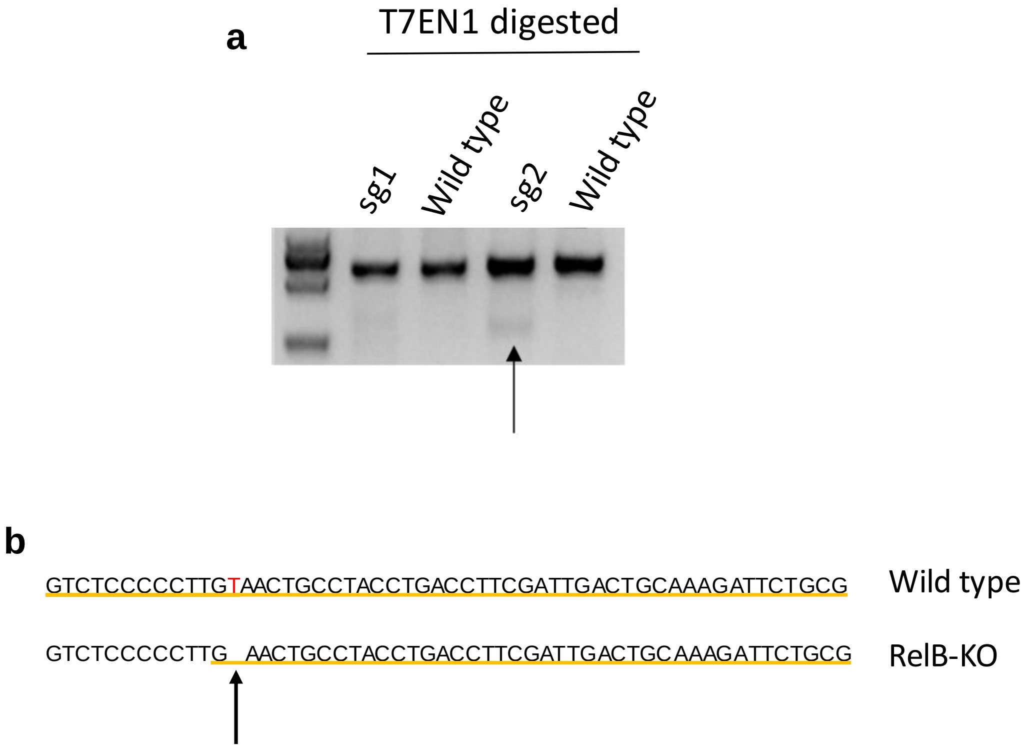

Fig. S1. Construction of RelB-knock out in TNBC cells. **a,** CRISPR-Cas9-based RelB-knock out in MDA-MB-231 cells was conducted using sg1 and sg2 targets. After cell transfected, the target regions were amplified by PCR and followed by T7EN1 digestion. An arrow indicates the potential gene editing. **b,** Multiple colonies were selected by DNA sequencing. Compared to the wild genotype, the knocked out genotype was generated by frame-shift deletion of a “T” indicated as an arrow.
