# Supplementary material for: RelB sustains endocrine resistant malignancy: an insight of noncanonical NF-κB pathway into breast Cancer progression
Source: Cell Commun Signal. 2020 Aug 17;18:128. doi: 10.1186/s12964-020-00613-x (PMC7430126; doi:10.1186/s12964-020-00613-x)
Supplement: Supplementary file 5 — Additional file 4. [file 12964_2020_613_MOESM5_ESM.pdf]

Additional file 4. Table S2:

Sequences of the primers used in RT-qPCR and ChIP-PCR

			Sequence (5'→3')
RT-qPCR	p21	Forward	TGTCCGTCAGAACCCATGC
		Reverse	AAAGTCGAAGTTCCATCGCTC
	p27	Forward	AACGTGCGAGTGTCTAACGG
		Reverse	CCCTCTAGGGGTTTGTGATTCT
	c-Myc	Forward	CAGCTGCTTAGACGCTGGATTT
		Reverse	ACCGAGTCGTAGTCGAGGTCAT
	CyclinD1	Forward	GCTGCGAAGTGGAAACCATC
		Reverse	CCTCCTTCTGCACACATTTGAA
	CyclinE1	Forward	AAGGAGCGGGACACCATGA
		Reverse	ACGGTCACGTTTGCCTTCC
	Bcl2	Forward	GGTGGGGTCATGTGTGTGG
		Reverse	CGGTTCAGGTACTCAGTCATCC
	Bcl-xL	Forward	GAGCTGGTGGTTGACTTTCTC
		Reverse	TCCATCTCCGATTCAGTCCCT
ChIP	MMP1	Forward	GACTACAGGTGCATGACTCCA
		Reverse	GTTAAGCTGCCTGGTACCCT
	GAPDH	Forward	GGAGCGAGATCCCTCCAAAAT
		Reverse	GGCTGTTGTCATACTTCTCATGG
