# Supplementary material for: RelB sustains endocrine resistant malignancy: an insight of noncanonical NF-κB pathway into breast Cancer progression
Source: Cell Commun Signal. 2020 Aug 17;18:128. doi: 10.1186/s12964-020-00613-x (PMC7430126; doi:10.1186/s12964-020-00613-x)
Supplement: Supplementary file 6 — Additional file 5. [file 12964_2020_613_MOESM6_ESM.pdf]

Additional file 5. Figure S2:

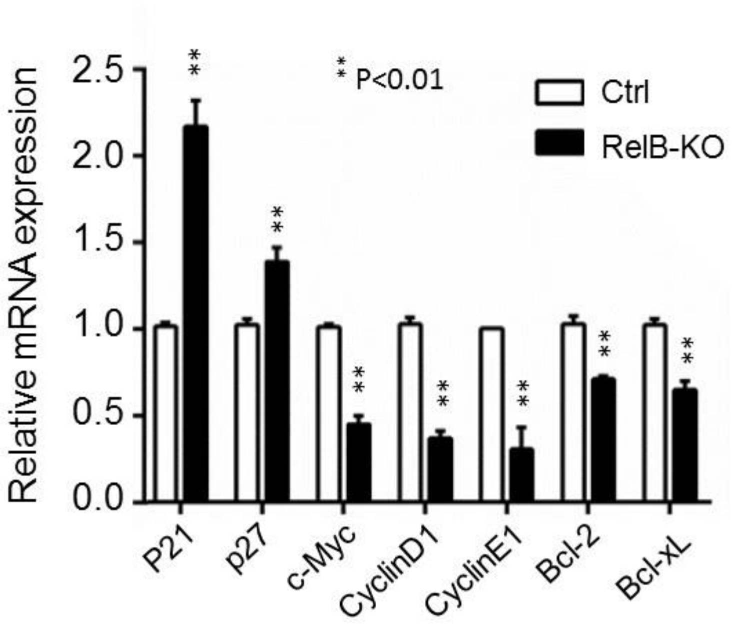

Fig. S2. The mRNA levels of cell cycle and apoptosis regulators. Total RNA was isolated from RelB-knocked out MDA-MB-231 cells and the parent cells. RT-qPCR was performed to determine the mRNA levels of relating genes.
