# Supplementary material for: RelB sustains endocrine resistant malignancy: an insight of noncanonical NF-κB pathway into breast Cancer progression
Source: Cell Commun Signal. 2020 Aug 17;18:128. doi: 10.1186/s12964-020-00613-x (PMC7430126; doi:10.1186/s12964-020-00613-x)
Supplement: Supplementary file 7 — Additional file 6. [file 12964_2020_613_MOESM7_ESM.pdf]

Additional file 6. Figure S3:

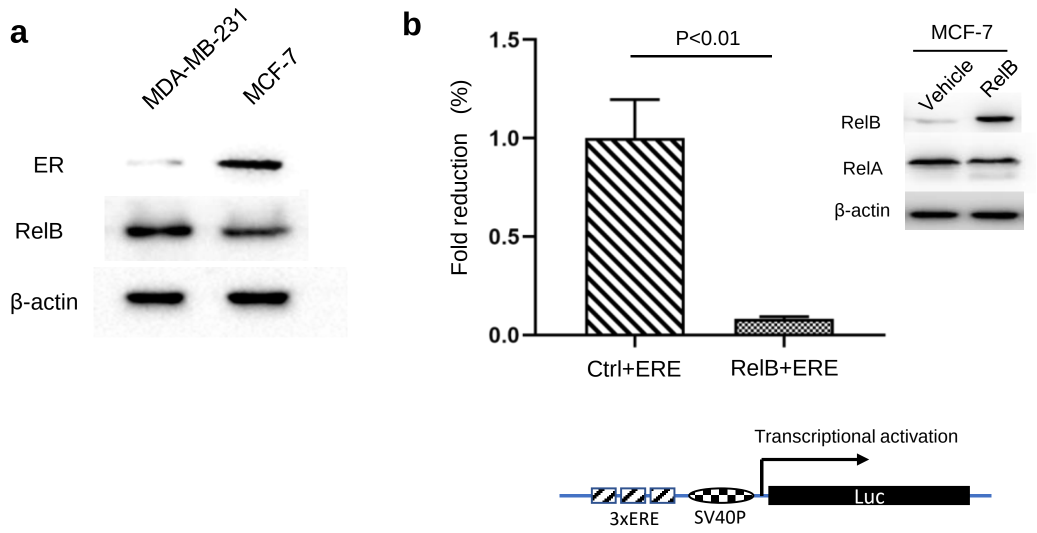

Fig. S3. The correlation of RelB and ER in BCa cells. **a,** The constitutive levels of RelB in ER-positive and ER-negative BCa cells. **b,** Three ER elements (3xERE) was cloned in SV40 promoter pGL3 (SV40P/pGL3) vector. The ER-driven luciferase construct was transfected into RelB-overexpressed MCF-7 cells and MCF-7 control cells, the reduction in the reporter activity by overexpression of RelB was plotted.
