# Supplementary material for: RelB sustains endocrine resistant malignancy: an insight of noncanonical NF-κB pathway into breast Cancer progression
Source: Cell Commun Signal. 2020 Aug 17;18:128. doi: 10.1186/s12964-020-00613-x (PMC7430126; doi:10.1186/s12964-020-00613-x)
Supplement: Supplementary file 8 — Additional file 7. [file 12964_2020_613_MOESM8_ESM.pdf]

Additional file 7. Figure 4:

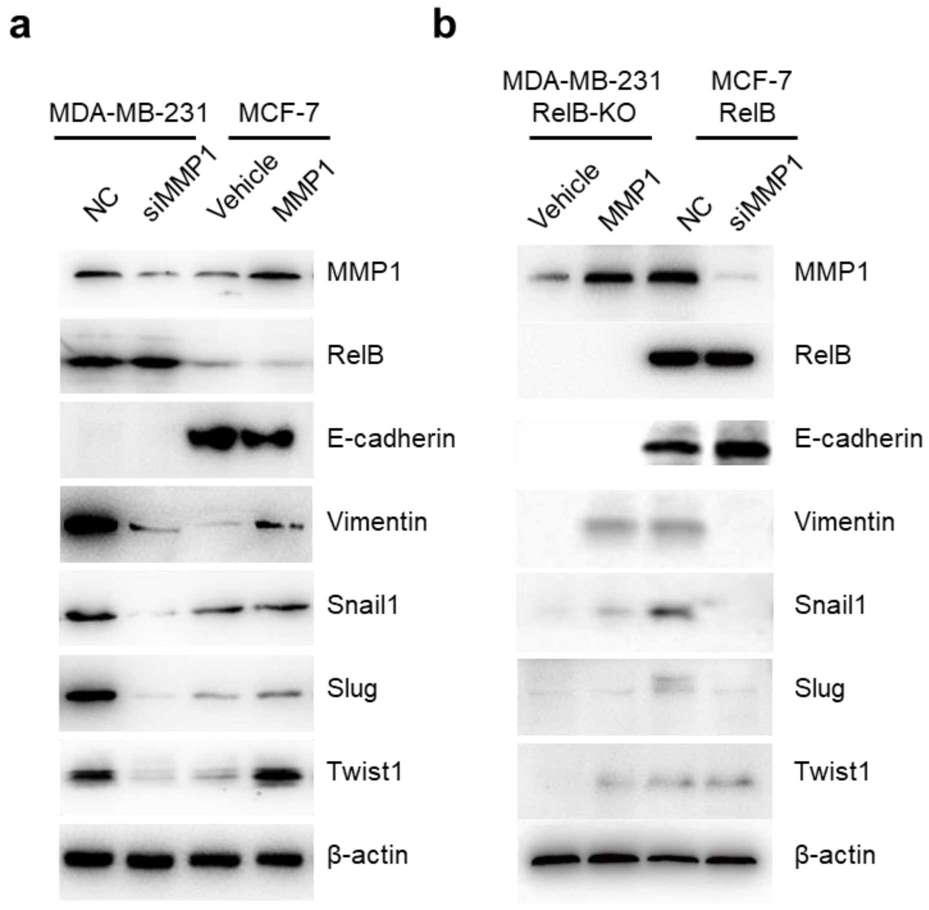

Fig. S4. The function of MMP1 in EMT activation**. a,** To examine whether MMP1 promotes EMT activation, MMP1 was either overexpressed in MCF-7 cells or silenced in MDA-MB-231 cells. **b,** To determine the effect of MMP1 on EMT activation under the RelB regulation, MMP1 was overexpressed in RelB-knocked MDA-MD-231 cells or silenced in RelB-overexpressed MCF-7 cells. The expression levels of EMT markers were measured by western blots.
